# Identification of the bacterial community that degrades phenanthrene sorbed to polystyrene nanoplastics using DNA-based stable isotope probing

**DOI:** 10.1038/s41598-024-55825-9

**Published:** 2024-03-04

**Authors:** Stephen Summers, Mohammad Sufian Bin-Hudari, Clayton Magill, Theodore Henry, Tony Gutierrez

**Affiliations:** 1 Institute of Mechanical, Process and Energy Engineering (IMPEE), School of Engineering and Physical Sciences, Heriot-Watt University, Edinburgh, EH14 4AS UK; 2 Singapore Centre for Environmental Life Sciences Engineering, Life Sciences Institute, National University of Singapore, Singapore, 119077 Singapore; 3 St John’s Island National Marine Laboratory, National University of Singapore, Singapore, 098634 Singapore; 4 Department of Isotope Biogeochemistry, Helmholtz Centre for Environmental Research-UFZ, Permoserstraße 15, 04318 Leipzig, Germany; 5 Institute for GeoEnergy Engineering, School of Energy, Geoscience, Infrastructure and Society, The Lyell Centre, Heriot-Watt University, Edinburgh, EH14 4AS UK; 6 School of Energy, Geoscience, Infrastructure and Society (EGIS), Heriot-Watt University, Edinburgh, EH14 4AS UK; 7 Department of Forestry Wildlife and Fisheries, Centre for Environmental Biotechnology, The University of Tennessee, Knoxville, TN 36849 USA

**Keywords:** Microplastics, Nanoplastics, Marine snow, Phenanthrene, DNA-SIP, Microbiology, Microbial communities, Environmental microbiology

## Abstract

In the Anthropocene, plastic pollution has become a new environmental biotope, the so-called plastisphere. In the oceans, nano- and micro-sized plastics are omnipresent and found in huge quantities throughout the water column and sediment, and their large surface area-to-volume ratio offers an excellent surface to which hydrophobic chemical pollutants (e.g. petrochemicals and POPs) can readily sorb to. Our understanding of the microbial communities that breakdown plastic-sorbed chemical pollutants, however, remains poor. Here, we investigated the formation of 500 nm and 1000 nm polystyrene (PS) agglomerations in natural seawater from a coastal environment, and we applied DNA-based stable isotope probing (DNA-SIP) with the 500 nm PS sorbed with isotopically-labelled phenanthrene to identify the bacterial members in the seawater community capable of degrading the hydrocarbon. Whilst we observed no significant impact of nanoplastic size on the microbial communities associated with agglomerates that formed in these experiments, these communities were, however, significantly different to those in the surrounding seawater. By DNA-SIP, we identified *Arcobacteraceae*, *Brevundimonas*, *Comamonas*, uncultured *Comamonadaceae*, *Delftia*, *Sphingomonas* and *Staphylococcus*, as well as the first member of the genera *Acidiphilum* and *Pelomonas* to degrade phenanthrene, and of the genera *Aquabacterium*, *Paracoccus* and *Polymorphobacter* to degrade a hydrocarbon. This work provides new information that feeds into our growing understanding on the fate of co-pollutants associated with nano- and microplastics in the ocean.

## Introduction

Marine plastic pollution is omnipresent and one of the most important environmental issues in this century. When plastics enter the sea from any number of sources, these materials fragment into smaller micro- (1000 µm to 5 mm) and, subsequently, nano-sized (< 1000 µm) particles that largely form the continuum of invisible marine plastic debris^1^. The ubiquitous presence of this plastic size range raises concerns over damage that it could potentially cause to individual marine species, whole ecosystems and to human health^2,3^. Due to their very tiny size, the detection and quantification of nanoplastics in any environment is still a major challenge. We know considerably more about microplastics (of size > 1000 µm), which with respect to the ocean, this plastic size range has been detected in virtually every environmental phase (water, sediments, sea ice, biota) from the Arctic to the Southern Oceans [^4^ and references therein]. Information on the distribution and quantities of nanoplastics in the ocean, however, remains a major knowledge gap and limited to only a few studies that have investigating this. For example, one full-depth (8–4369 m) survey of the Arctic Central Basin found an average abundance of 0.7 particles m^−3^ of size range 250 µm to 5 mm^5^. In another study, 42–6595 of plastic particles Kg^−1^ were identified in deep-sea sediments (2340–5570 m) of the Arctic, with 80% of the particles found to be < 25 µm in size^6^. Importantly, this study suggested that removal of microplastics from the surface ocean to the abyss does take place. A more recent study examining the combined mass of the three most-littered plastics (polyethylene, polypropylene, polystyrene) of size range 32–651 um in the open Atlantic Ocean (following a transect from north to south) found a staggering 11.6–21.1 million tonnes in only the top 200 m^4,5,7^.

Considering the large surface area-to-volume ratio of nano- and micro-sized plastics, and the enormous quantities of these particles that are predicted to lie hidden in the global ocean, they are likely to play a significant role in the partitioning and fate of other hydrophobic chemical pollutants, such as polycyclic aromatic hydrocarbons (PAHs), polychlorinated biphenyls (PCBs) and other persistent organic pollutants (POPs) in the marine water column and sediment^8,9^. This is because these types of chemical pollutants have high binding (*K*_*d*_) coefficients^10^ which renders them with a high capacity to adsorb to hydrophobic plastic polymers (the most common plastics produced commercially), often via hydrophobic or π–π interaction mechanisms^11,12^. For example, phenanthrene has been reported on plastics at concentrations reaching 16.4 mg/kg, whereas phthalates and polychlorinated biphenyls (PCBs) have been reported at concentrations of up to 84.6 mg/kg and 18.6 mg/kg, respectively^13^. These types of co-pollutants can in turn influence the microbial colonisation and development of biofilms on the plastic surface—the zone defined as the ‘plastisphere’^14^. Sequencing surveys of the plastisphere have revealed hydrocarbon-degrading microorganisms (comprising up to 34.4% of total plastisphere communities) that can metabolize PAHs and other POPs^15^. The most common taxa reported comprise members affiliated to the Euryarchaeota and Proteobacteria, with *Oceaniserpentilla* and *Celeribacter* the most common species found in the plastisphere^16,17^.

One mechanism by which nanoplastics, and potentially the smaller-size fraction of microplastics (< 1000 µm), are removed from the marine water column is via their incorporation into marine snow, resulting in plastic agglomerations that, when no longer buoyant, may then sink and become transported to the sea floor^18–20^. Previous work by our group showed nano- and microplastic particles to almost spontaneously agglomerate together with exopolysaccharide substances (EPS) in seawater and become colonised by microorganisms^19^. Other studies have similarly shown nano- and microplastic particles to associate with free-floating ‘gel’ particles, such as marine snow, and to develop a rich microbial community^18,19,21,22^. The biodegradation of nano-/microplastic-associated co-pollutants under environmentally relevant conditions—i.e. when these pollutants are naturally bound within a marine exopolymer matrix, such as marine snow—remains unknown. Reminiscent to marine snow, plastic agglomerations will also appear as an appealing morsel of food to any number of marine organisms, though the jury is still out on whether the nano-/microplastic particles themselves cause deleterious effects to organisms that have ingested them^23,24^. Such effects have been linked to the toxic co-pollutants, like PAHs and other POPs, that are found adsorbed to the plastic particles^23,25,26^, suggesting that the plastic particles are acting as concentration hotspots of these toxic pollutants and as vehicles for their entry into the food chain.

Considering that microorganisms are at the heart of removing toxic chemical pollutants from the marine environment by transforming them into less harmful intermediates or end products, we hypothesise that the microorganisms associated with nano- and microplastic agglomerates play an important role in the breakdown of toxic co-pollutants that can become sorbed on their surface. To the best of our knowledge, no study has so far explored the plastisphere relating to POP degradation, or which specific additives may be used as a source of carbon by microorganisms. Here, we examined the formation of nano- and microplastic agglomerations in natural seawater from a coastal environment, and we applied DNA-based stable isotope probing (DNA-SIP) with isotopically-labelled phenanthrene sorbed to the plastic particles in order to identify the members of the microbial community capable of utilising this hydrocarbon as a carbon source. We chose phenanthrene because it is commonly used as a model chemical pollutant, and it is found as a plastic additive^27^ and also known to readily sorb onto plastics^28^. Polystyrene (PS) particles of spherical diameters 500 nm and 1000 nm were custom synthesized, and their surface primed with the ^13^C-labelled phenanthrene. Using this approach, several bacterial taxa were identified within plastic marine snow aggregates that are capable of degrading the plastic-sorbed phenanthrene, including some that had, hitherto, not previously been reported to utilise this compound as a carbon source. This work provides new information that feeds into our growing understanding on the fate of co-pollutants associated with nano- and microplastics in the ocean.

## Results

### Plastic particle size and surface characteristics

The sizes of the two custom-synthesized PS particles (500 nm and 1000 nm) were analysed by a DLS Zetasizer Pro or Mastersizer2000 analyser and found to measure 505.5 ± 50.3 nm and 1434.0 ± 7.8 nm, respectively. Initial ζ-potential measurements showed that the particle surface charge for the 500 nm and 1000 nm particles was, respectively, − 51.8 ± 3.3 mV and − 38.3 ± 3.5 mV. Following removal of any contaminants, such as surfactants, from the plastic particle suspensions, the ζ-potential measurements were, respectively, − 36.3 ± 0.2 mV and − 12.7 ± 1.2 mV.

**Figure 1:**
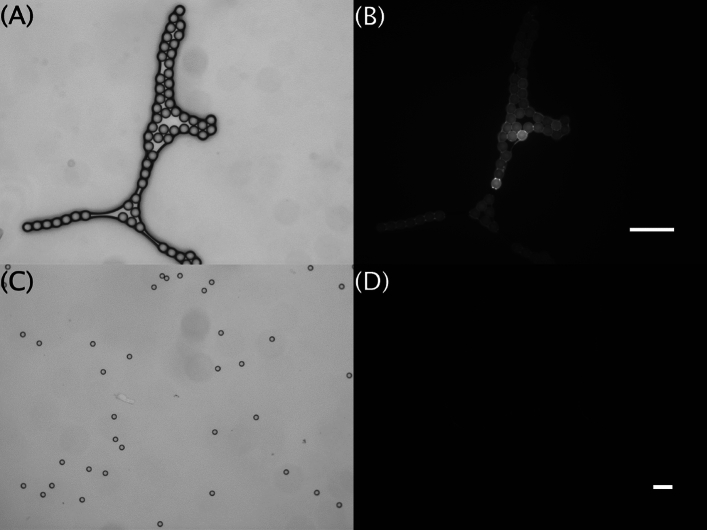
Polystyrene nanoplastic 1000 nm particles observed under the light (**A**, **C**) and epifluorescence (**B**, **D**) microscope before and after exposure of the particles to phenanthrene. Particles exposed to phenanthrene (**A**, **B**) have a tendency to agglomerate, and the adsorption of the phenanthrene shows up well under epifluorescence (**B**). Particles not exposed to phenanthrene (**C, D**; non-exposed controls) showed no visible presence of phenanthrene on the surface of the particles, especially when viewed under the epifluorescence microscopy (**D**). Scale bars, 5 µm.

The presence of adsorbed phenanthrene on the surface of the 500 nm and 1000 nm nanoplastic particles was confirmed by GC–MS. This was also confirmed by fluorescence microscopy for the larger 1000 nm particles (Fig. 1). As expected, no fluorescence was observed on the particles that were not exposed to phenanthrene (Fig. 1). The PDI of the two plastic particle sizes did not alter significantly due to the presence of adsorbed phenanthrene. The PDI of the 500 nm particles was the lowest (at ca. 0.1) measured of the two particle sizes, indicating this particle size was likely well dispersed in solution. In comparison, the 1000 nm particles had PDI values > 0.5, indicating this particle size was most likely heterogeneously distributed (i.e. a mix of dispersed and agglomerated particles) in solution. The ζ-potential readings were − 26.2 ± 1.0 mV and − 8.4 ± 0.7 mV for, respectively, the 500 nm and 1000 nm particles with adsorbed phenanthrene.

### Formation of nanoplastic agglomerates and their associated bacterial communities

**Figure 2:**
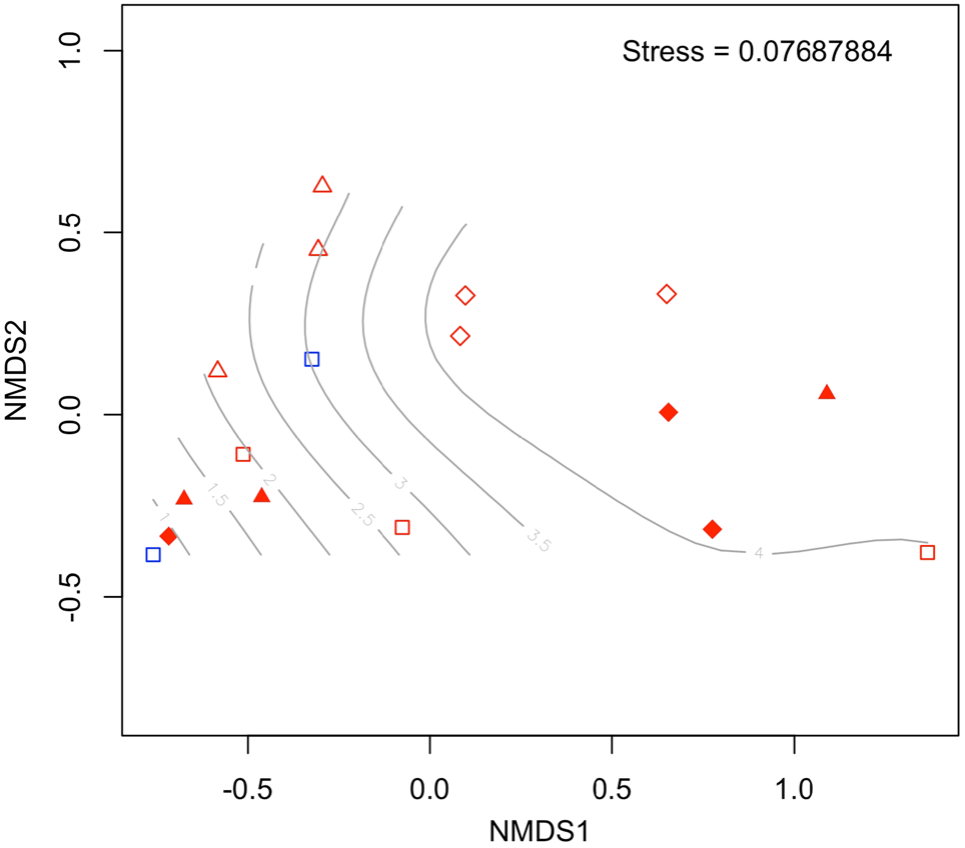
Non-metric multi dimensional scaling (nMDS) plot showing the dissimilarity between bacterial communities for each of the Firth of Forth (FoF) samples analysed. Symbols in red represent the communities associated with agglomerations; symbols in blue are background water controls. Closed symbols represent communities from incubations with nanoplastics with sorbed phenanthrene; open symbols represent communities from incubations that did not contain phenanthrene. Seawater controls (squares), 500 nm nanoplastics (triangles) and 1000 nm nanoplastics (diamonds) are indicated. Light grey contour lines indicate H′ diversity.

**Figure 3:**
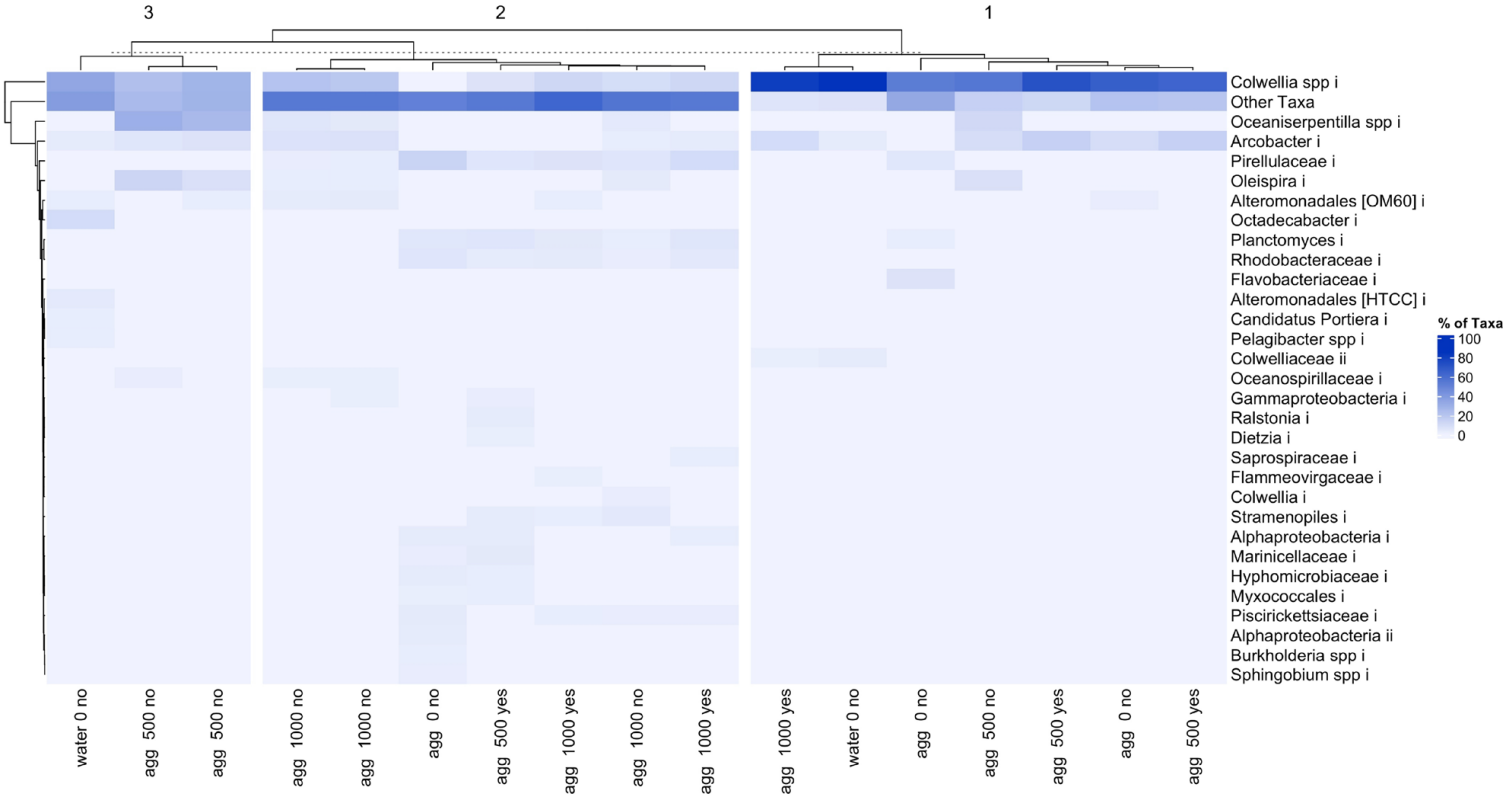
Heatmap showing the relative abundance of the major taxa (> 2% relative abundance) in incubation experiments with seawater from the Firth of Forth (FoF). X-axis labels describe ‘matrix-plastic size-presence of phenanthrene’. The blue scale represents the abundance of taxa, from highly abundant (dark blue) to less abundant (light blue). The heatmap is separated into three columns to represent the three possible influencing factors—seawater only/agglomerate formation (water/agg), nanoplastic presence/absence and size [0 (not present)/500/1000), and presence/absence of phenanthrene (yes/no)]. Columns and rows are separated based on Pearson correlation co-efficient.

Visual observation of the vials showed the formation of agglomerates in all the treatments and natural seawater controls (no plastic added). Agglomerates were ~ 0.5 mm in size and appeared dark brown. Barcoded 16S rRNA Illumina MiSeq paired-end sequencing of the bacterial community associated with the agglomerates indicated a Shannon–Weiner diversity (H′) ranging from 0.7 to 4.4 (Fig. 2). Using plastic size and presence/absence of phenanthrene as treatment factors, Permanova analyses did not indicate any significant effect by either of these factors on the bacterial community (phenanthrene: Permanova, F_1–16_ = 1.03, R_2_ = 0.06, *p* = 0.184; Plastic size: Permanova, F_1–16_ = 2.06, R_2_ = 0.12, *p* = 0.096). There was also no significant interaction identified between these treatment factors. From these incubations, DNA extracted from the water surrounding the agglomerates failed to be sequenced. From the 16S rRNA gene sequencing analysis of the agglomerates, 803 individual SNVs were identified across all treatments. Of these, 77 SNVs were either significantly different between treatments, or showed some form of statistically significant interaction between treatments. The most abundant taxa observed across all treatments were SNVs identified as *Colwellia* (35%), *Arcobacter* (7%), *Oceaniserpentilla* (5%), *Pirellulaceae* (4%), *Oleispira* (3%), and *Planctomyces* spp. (3%) (Fig. 3). The most abundant taxa within treatments exposed to phenanthrene were *Colwellia* (42%), *Arcobacter* (8%), *Pirellulaceae* (4%), *Planctomyces* (3%), and *Rhodobacteraceae* (2%). The most abundant taxa identified in agglomerates that formed in incubations with only the nanoplastic (no phenanthrene) were *Colwellia* (34%), *Arcobacter* (7%), *Oceaniserpentilla* (7%), *Pirellulaceae* (7%) and *Oleispira* (3%).

### SIP-identified phenanthrene-degraders associated with nanoplastics

**Figure 4:**
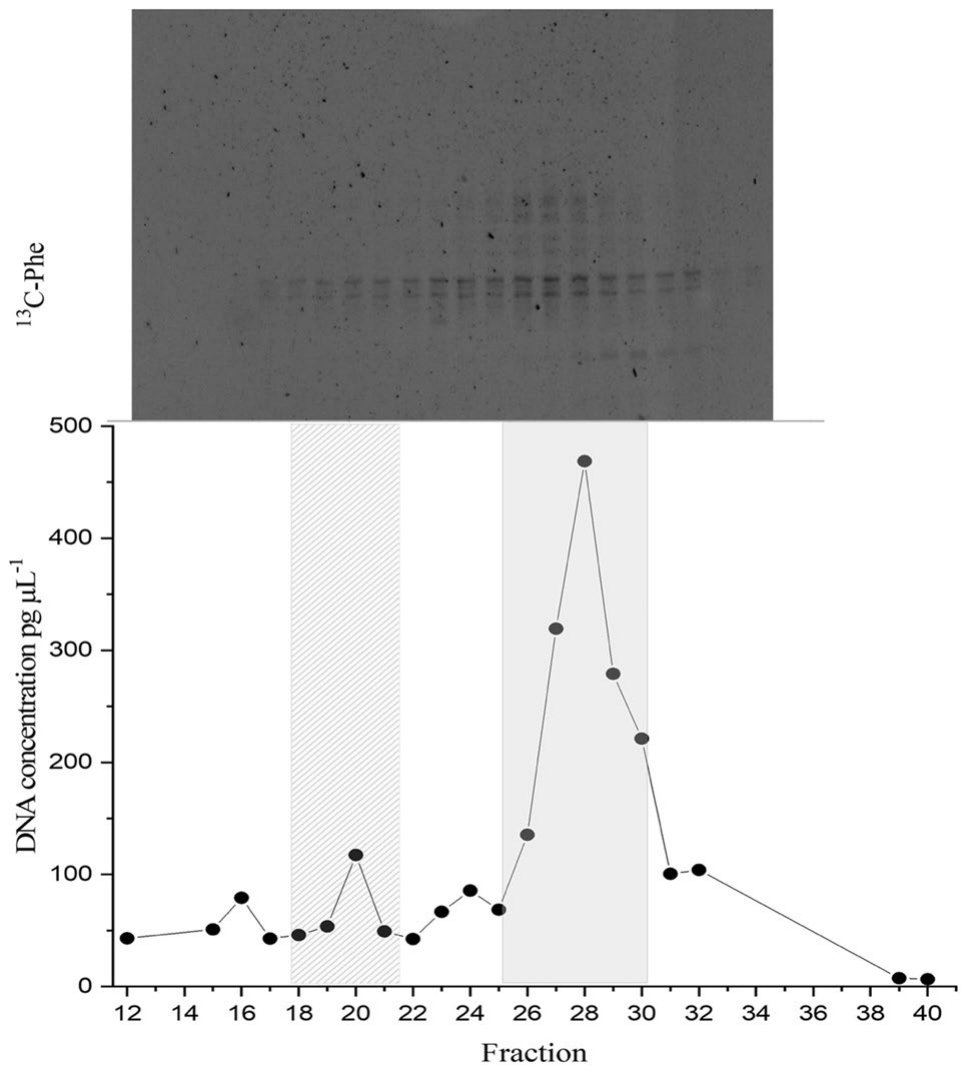
Distribution of the ‘heavy’ and ‘light’ DNA in separated SIP fractions. The top panel shows the DGGE profile of bacterial PCR products from separated [^13^C]-phenanthrene fractions (12 to 34) with decreasing densities from left to right. The distribution of qPCR-quantified total 16S rRNA gene sequences in fractions from the [^13^C]-phenanthrene incubation is shown below the DGGE image (black filled circle). Fractions 18–21 (left shaded area) were determined to represent ^13^C heavy DNA and were combined for further analysis. DGGE banding patterns for a given fraction are aligned with the corresponding gene abundance data below.

Day 7 was selected as the endpoint of the SIP experiment for extraction of DNA from ^13^C incubations because complete removal of the phenanthrene in the unlabelled (^12^C) incubations, based on GC–MS analysis of subsamples taken from these incubations, occurred at day 5 (results not shown). DNA extractions were performed on each of the duplicate ^13^C incubations at day 7 for subsequent isopycnic ultracentrifugation to isolate the ^13^C-enriched ‘heavy’ DNA for analysis. DGGE analysis of the fractions derived from the labelled and unlabelled incubations showed clear evidence of isotopic enrichment of DNA in ^13^C-phenanthrene incubations, separation of ^13^C-labeled and unlabelled DNA, and different banding patterns between the ^13^C-enriched and unenriched DNA fractions (Fig. 4). From the banding pattern for the ^13^C-incubation, fractions 18–21 were combined and the community representing this isotopically-labelled DNA was sequenced by Nanopore long read sequencing to obtain almost full length 16S rRNA gene sequences. We note that this subset of four fractions represents a small peak in the total DNA concentration of the nanodrop profile. This we assume is likely because the adsorbed phenanthrene would have been less, though not incompletely, amenable for uptake and biodegradation by members of the microbial community that could use it as a sole carbon source (discussed below).

**Figure 5:**
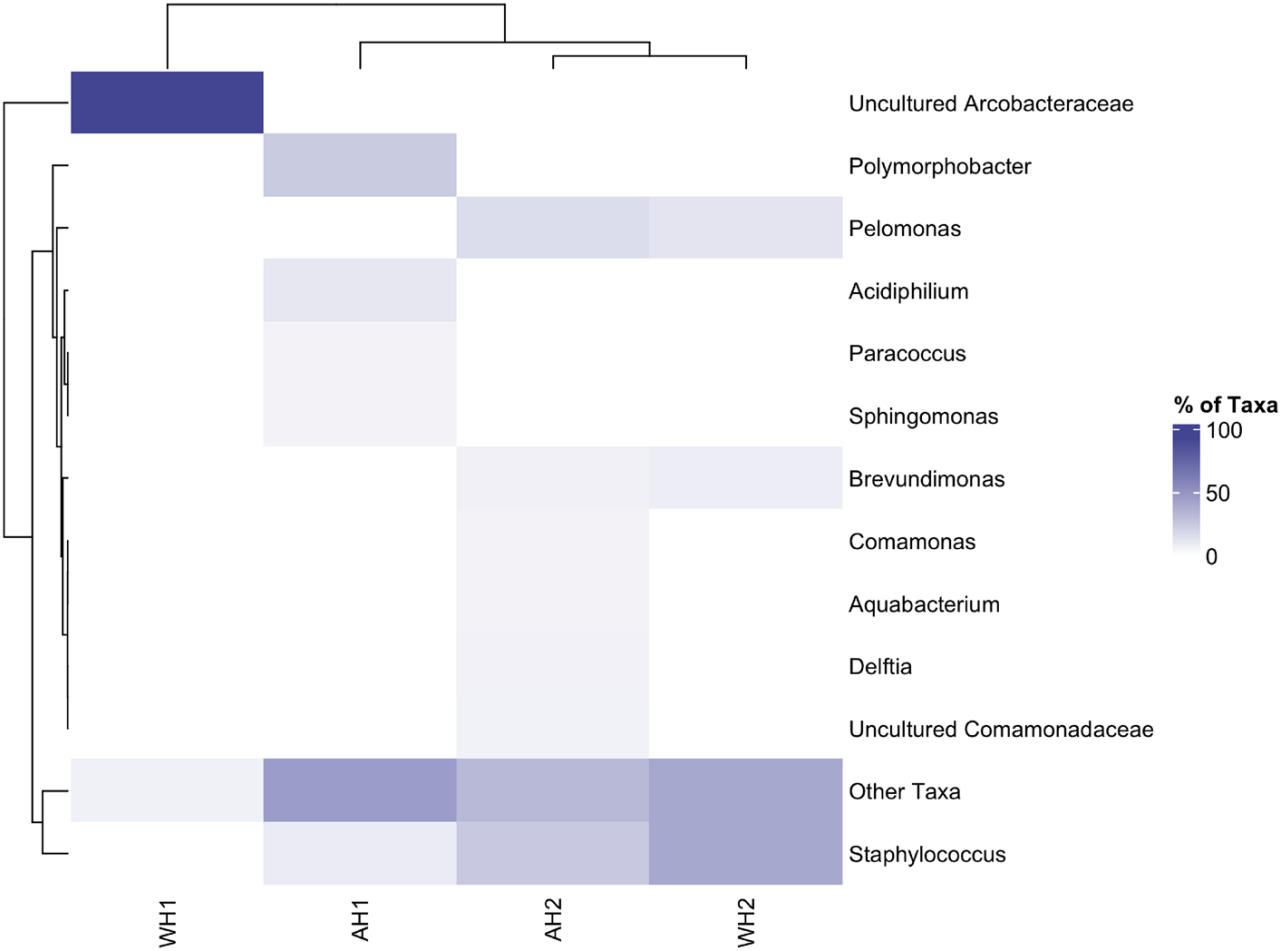
Relative distribution of taxa found in the heavy fractions from the SIP incubations with seawater from the Firth of Forth (FoF) exposed to polystyrene nanoparticles of size 500 nm primed with ^13^C-labelled phenanthrene. AH1 and AH2 represent agglomerate samples from the duplicate SIP incubations; WH1 and WH2 represent the corresponding samples of the water surrounding these agglomerates. The colour scale represents the relative abundance (%), from highly abundant (dark; 100%) to undetectable (white; 0%).

Near full-length 16S rRNA gene sequences (average length of 1431 bp) were obtained from the isotopically-labelled heavy fractions derived from agglomerates and from the surrounding seawater fractions in these incubations, resulting in 94 unique sequences (Supplementary Table S1). Thirteen taxa were found to dominate the isotopically-labelled community associated with agglomerates or the free-living community (*i.e.* seawater surrounding the agglomerates) (Fig. 5). The most abundant taxon, representing 94% of total sequences, was an uncultured *Arcobacteraceae* identified in the water surrounding the agglomerates from one of the duplicate incubations. Other major taxa identified in isotopically-labelled DNA (with > 5% representation) were: *Staphylococcus* (in agglomerations from both duplicate SIP incubations, and in the surrounding water from only one of these incubations); *Polymorphobacter*, *Delftia*, *Acidiphilium*, *Comamonas*, uncultured *Comamonadaceae*, *Aquabacterium*, *Paracoccus* and *Sphingomonas* (in agglomerations from one of the duplicate SIP incubations); and *Pelomonas* and *Brevundimonas* (in agglomerations and surrounding water from one of the duplicate SIP incubations). Notably, the community identified with the nanoplastic-adsorbed phenanthrene (*i.e.* identified associated with the agglomerations recovered from the SIP incubations) was found to be more diverse than that in the surrounding water.

Supplementary Table 2 shows the relative abundance of the various taxa identified in the ^13^C-enriched community compared to those found in the ^12^C-unlabelled community of the nanoplastic agglomerates and surrounding seawater. Well-established taxa of phenanthrene-degrading bacteria were solely confined to the ^13^C-enriched community. We used this as an additional guide to the DGGE banding and nanodrop DNA concentration profiles in order to more robustly confirm that any of the taxa in the ^13^C-labelled DNA were capable of phenanthrene degradation. For this, we applied a ≥ 1% relative abundance detection limit for taxa found in either of the two SIP incubations, and for which were at least fivefold higher in relative abundance in the ^13^C-enriched community compared to in the corresponding duplicate ^12^C-unlabelled community. From this, we identified *Staphylococcus*, *Pelomonas*, *Polymorphobacter*, *Acidiphilium*, *Paracoccus* and uncultured *Rhodobacteracea* as phenanthrene degraders.

## Discussion

Using the DLS Zetasizer Pro and Mastersizer2000 analyser showed that the 1000 nm commercial PS particles to be ~ 40% larger than prescribed by the manufacturer, but examination of these (and the 500 nm particles) under the microscope showed that both particle sizes were as prescribed. The overestimated size measurements for the 1000 nm particles may be explained, in part, by the large PDI values measured, and indicates the agglomeration of this larger particle size. For each of the two particle sizes, the variability in the ζ-potential at the pre-wash stage is reflected in the variability of the PDI, with 500 nm having the lowest values compared to the 1000 nm particles which had ζ-potential values closer to zero mV. It was apparent that the wash step decreased the negative charge of the plastic particle surface, and the addition of the phenanthrene to the surfaces of these particles further decreased the negative surface charge down to values approaching − 10 mV. These results also indicate that the particles, and most especially the larger (1000 nm) particles, formed agglomerates from the start of our seawater incubation experiments. Moreover, the changes in zeta potential may have environmentally relevant consequences, as one study showed that the inclusion of PS nanoplastics can also impact the zetapotential of bacterial cell membranes^29^.

Some studies have reported different communities associated with marine suspended agglomerations (e.g. marine snow or other suspended particulate organic matter) to those found in the surrounding water^30,31^. Here, we assessed this for agglomerations formed in a coastal environment with PS nanoplastics of two particle sizes with/without sorbed phenanthrene. However, it should be noted that this experiment was conducted using PS particles generated in the laboratory, which are unlike nanoplastics that are found in the natural marine environment, the latter of which will have undergone natural weathering processes. With that in mind, we found the bacterial community of agglomerates formed with nanoplastics of the 500 nm particle sizes was not different to those formed with the 1000 nm particles, and in both cases the communities were distinct from the microbial communities in the surrounding water (Fig. 2). It can be seen from Fig. 2 that the agglomeration with the smaller 500 nm PS particles were dominated by *Colwellia* spp., whereas the larger 1000 nm PS appears to have had a greater influence on the rare biosphere. PS nanoplastics have been reported to cause toxicological effects to some bacterial species, which may explain this differential enrichment of some taxa on the plastic particles compared to the community in the surrounding seawater. For example, Sun et al.^32^ showed the growth rates of *Halomonas alkaliphile* were significantly reduced in the presence of nanoplastics, whereas larger-size microplastics, of the same polymer type, had a lesser impact on this bacterial species. The authors hypothesised that reactive oxygen species are generated by nanoplastics, subsequently inducing oxidative stress on *H. alkaliphile*. Indeed, the generation of these reactive oxygen species and the resulting stress has been observed in several studies to date that employ nanoplastics^33–35^, affecting both prokaryotes and eukaryotes. Translating this to our observations and to how nano and microplastics could impact microbial communities and their association with marine snow particles in the ocean, smaller plastic particle sizes have higher surface areas, likely increasing their propensity to generate and release reactive oxygen species, or even other toxic leachates, and as a consequence resulting in growth inhibition to certain species of bacteria. We note that our study did not employ the same amine modifications on the nanoplastic surface which Sun et al.^32^ used in their study, but we believe this would not discount this as a plausible explanation, especially also taking into account phenanthrene as an inhibitor of some bacterial species. The significant effect of nanoplastic size, both in control and phenanthrene-adsorbed treatments, has also been observed with planktonic eukaryotic organisms, such as *Daphnia* spp., where 50 nm plastics were found to inhibit growth of these organisms, whereas larger plastic sizes had no apparent effect^36^. Other studies have shown the presence of phenanthrene to stimulate the formation of reactive oxygen species in several biological systems^37,38^. We conjecture that by this mechanism, the combination of phenanthrene adsorbed onto nanoplastics may have contributed to structuring the bacterial communities observed in our experiments by inhibiting the activities of some taxa, especially in the case of smaller-sized plastics (as observed with the 50 nm PS particles), whilst enriching for other taxa, such as those that can use phenanthrene as a carbon and energy source (discussed below).

The most abundant taxa identified in treatments with either PS nanoplastic particle size (500 nm or 1000 nm) were *Colwellia*, *Arcobacter*, *Oceaniserpentilla*, *Pirellulaceae*, and *Oleispira*. All of these genera have been reported to contain members possessing hydrocarbon-degrading qualities, or that have been reported enriched when exposed to hydrocarbons^39–41^. While several hydrocarbon-degrading microorganisms have been implicated in PS degradation^42^, we considered analysing for the degradation of the PS. However, the rate at which this occurs is known to be very slow because PS is deemed one of the more recalcitrant plastics to microbial degradation. As such, monitoring microbial degradation of the PS in a relatively short-term experiment, as in our SIP experiment, was met with quite a challenge at the time and, thus, was aborted. The marine snow agglomerates that formed with 500 nm or 1000 nm nanoplastics sorbed with phenanthrene were dominated by members of the *Alteromonadales* (closest match to J115 based on a search in the Greengenes database). This order include genera with hydrocarbon-degrading capabilities which have been reported to become enriched when exposed to crude oil or its refined products^40,43^—chemicals which, as mentioned, are akin in structure and composition to many plastic polymer types, like PS. The order also includes species that can produce EPS that can effect the formation of marine agglomerations, including in the presence of hydrocarbons^44^. Although *Alteromonadales* were found dominant in our experiments, they were not enriched following the addition of nanoplastics with/without phenanthrene. *Oceaniserpentilla* (closest match to *O. haliotis*)^45^ was found enriched more in agglomerates that formed in the treatments with nanoplastics without sorbed phenanthrene. This genus sits within the order *Oceanospirillales* which contains most of the known genera and species of obligate and generalist hydrocarbon degraders^46,47^. Hydrocarbon and/or PS degradation remains to be checked in the cultured relatives *O. haliotis*^45^, but one report showing enrichment of this genus during the Gulf of Mexico oil spill^48^ suggests this metabolic capability may be possible by these organisms. Nanoplastic particle size (500 nm or 1000 nm) and presence/absence of phenanthrene did not appear to have a pronounced impact upon the bacterial communities. Whilst we are unable to account for this, one possible reason may be because the microbial communities are already enriched by petrochemicals in the FoF, as it is a coastal area with a history of substantial industrial pollution from shipping, industrial discharge, commercial and recreational water activities, and drainage of surface road runoff^49–51^.

Following from our experiments showing that hydrocarbon-degrading bacteria will readily associate with nanoplastic agglomerations in seawater, using DNA-SIP we showed that phenanthrene adsorbed to PS nanoparticles can be actively degraded by certain members of the community, as evidenced by the uptake of the ^13^C-label into their DNA. Of those identified in this study, some *Staphylococcus* sp. have been found to degrade hydrocarbons^52^, and members of the genus *Paracoccus* have been reported enriched in marine biofilms exposed to oil pollution^53^. We also identified uncultured *Rhodobacteraceae* in the ^13^C-labelled community, which is a family comprising well known genera, such as members of the *Roseobacter* clade which are widely recognised for possessing multiple ring-cleavage pathways for degrading PAHs^54^. Whilst members of the genus *Acidiphilum* have been associated with hydrocarbon degradation in studies showing their enrichment in petrochemical polluted environments^55^, or to encode genes for the degradation of hydrocarbons^56^, the present study is the first to show these organisms are also capable of degrading phenanthrene. We also identified members of the genera *Polymorphobacter* and *Pelomonas*, which showed enrichment in the ^13^C treatments when compared to their representation in the ^12^C treatments. To our knowledge, no taxon within either of these two genera has previously been reported with the ability to degrade hydrocarbons, with the exception of a recent study describing a *Pelomonas* to utilise ethylbenzene as a sole carbon and energy source for growth^57^. Other taxa that were found in the ^13^C-labelled community of the PS agglomerates or surrounding seawater environment, and which have been reported with hydrocarbon-degrading qualities, were *Sphingomonas*^58^, *Brevundimonas*^59^, *Comamonas*^60^, *Aquabacterium*^61^ and *Delftia*^62^. All of these genera, with the exception of *Aquabacterium*, have been reported with the ability to degrade phenanthrene. Whilst any of these organisms may had contributed to the degradation of the phenanthrene adsorbed to the PS nanoplastic particles, we excluded them because, based on the screening process we used, they were not sufficiently enriched in the ^13^C-labelled community.

Our findings show that, when naturally embedded within marine snow agglomerations, nanoplastics sorbed with phenanthrene can enrich for a diverse community of hydrocarbon-degrading bacteria, and that these agglomerations, which are akin to marine oil snow particles^63,64^, could be acting as hotspots for organic pollutant microbial degradation. Access to the nanoplastic-sorbed phenanthrene by these bacteria is complicated by factors, such as, how tightly the phenanthrene is sorbed onto the plastic surface. This is because phenanthrene and other PAHs in general have high binding (*K*_*d*_) coefficients^10^ which renders them with a high capacity to adsorb to hydrophobic plastic polymers—often mediated via hydrophobic or π–π bonding of these chemicals^11,12^. In our experiments, as in natural marine waters, this will affect the sorbed-phenanthrene's bioavailability and biodegradation, which is likely to be much reduced compared to when phenanthrene molecules are freely-solubilised in seawater. Taking this into consideration, the sorbed phenanthrene may not have been sufficiently available for microbial degradation, and which could explain the small ^13^C-enriched total DNA peak represented by the subset of four fractions (18–21) shown in the nanodrop profile (Fig. 4).

Whilst plastic pollution and its degradation is pervasive in the global ocean and occurring even in cold marine environments^65^, this is mainly associated with less persistent or recalcitrant plastic polymer types that are more degradable than the one (i.e. PS) employed in this study. The microbial biodegradation of PS is possible, but the long chain polymer is relatively resistant to biodegradation, and as such this is not commonly reported. Sekhar et al.^66^ reported that PS components from retired electronic devices can act as a carbon source and sustain several bacterial taxa, such as *Brevundimonas* which we identified in our study by DNA-SIP to utilise phenanthrene sorbed to nanoplastics within marine agglomerates. Further work to explore the ability of this and other taxa to degrade PS and other relatively recalcitrant plastic polymers, such as by using SIP with ^13^C-labelled PS, will help shed new light on the biodegradability of these types of plastic polymers in the ocean and the microbial communities that can do this.

This study shows that nanoplastics with sorbed organic pollutants, such as phenanthrene, has little effect on the bacterial community associated with nanoplastic agglomerates. Notably, our findings allude to the potential for nanoplastic particles in altering the microbial communities associated with marine snow. This could be quite important, especially in the context of the 'biological pump' and 'microbial loop', as these processes profoundly regulate the sequestration of carbon and its remineralisation, and that of other nutrients, in the marine water column, and which are inextricably linked to climate-associated changes. The fact that nanoplastic particles associated with marine snow positively enriches for hydrocarbon-degrading and EPS-producing bacteria, even in the absence of plastic-sorbed petrochemicals, is intriguing as it suggests that nanoplastics in the marine water column might change the dynamics and metabolic processing of petrochemicals and other organic substances. An important question to address is how nanoplastic particles with sorbed co-pollutants and within marine snow agglomerates might impact higher organisms, from macroplankton to small and larger fish and other marine animals, should they ingest these types of agglomerates? It is possible that the agglomerates pass through the organisms unchanged and with no aberrant effects. Or, as might be more likely to occur, the phenanthrene (and other plastic-adsorbed organic pollutants if present), desorb and enter inside the tissues of the consumer, potentially leading to acute illness or chronic long-term disease^10,67^. Conversely, as a recent study has shown, the transfer of chemical pollutants by plastic debris may occur via a biphasic mode^24^, whereby the plastic material may equally, and just as easily, adsorb pollutants from the ingesting organism during its passage through the gut, thereby providing some sort of "internal cleansing" benefit. Using DNA-SIP with ^13^C-labelled phenanthrene sorbed to nanoplastics, our work posits that hydrocarbon-degrading bacteria should help mitigate the deleterious effects that these particles can have upon organisms that ingest them^9,68^.

## Methods

### Field sampling

Seawater was collected in March of 2017 from Fisherrow Harbour (55°56′42″N, 3°03′56″W) in the Firth of Forth (FoF) estuary—a site located near Edinburgh in Scotland. Briefly, several litres of surface seawater from this location were collected using a handpump before transportation to the laboratory. The site is located to nearby pollution sources such as sewage treatment and industrial activities, including inflows of surface runoff from land. Immediately upon collection, the seawater was placed in a cooler with ice, returned to the laboratory on the same day and used within 24 h for subsequent experiments. The waters collected had a surface temperature and salinity of 7 °C and 33.5 PSU, respectively.

### Synthesis and characterisation of 500 nm nanoplastic particles

Styrene (Fisher Scientific, UK) was used to synthesize spherical PS nanoparticles of size 500 nm and 1000 nm, as described by following previous procedures of Al-Sid-Cheikh et al.^69^, Hong et al.^70^. As the behaviour of nanoparticles can be influenced by their shape, spherical nano-PS particles were prepared to be comparable with most previous laboratory-based studies investigating some biological process with these particles^71^. Unreacted monomer or other chemicals were removed by ultra-filtration (exclusion size of membrane: 30,000 g mol^−1^). Particle morphology was observed by transmission electron microscopy (TEM; JEOL 1400) at an accelerating voltage of 80 kV. When not in use, the plastic particles were stored in sterilized deionised water (sdH_2_O). Characterization of the plastic particles to confirm their size was performed used a Mastersizer2000 (Malvern Panalytical ltd, Malvern, UK). In addition, the surface of the particles was examined with a DLS Zetasizer Pro (Malvern Panalytical ltd, Malvern, UK) to measure the ζ-potential. For these analyses, any ingredients (e.g. Tween 20) used for storage of the plastic particles were firstly removed by several washings (10,000×*g* for 4 h at 20 °C) with a phosphate-buffered saline solution (137 mM NaCl, 2.7 mM KCl, 10 mM Na_2_HPO_4_ and 1.8 mM KH_2_PO_4_). The washed plastic particles were then resuspended in sdH_2_O and analysed to confirm their size, shape and distribution, and their ζ-potential. In addition, the polydispersive index (PDI) was measured using the DLS Zetasizer Pro, and again also after priming the plastic particles with ^13^C-labelled phenanthrene (described below).

### Priming the nanoplastic particles with ^13^C-labelled or unlabelled phenanthrene

The custom-synthesized nanoplastic particles were primed with either unlabelled phenanthrene or uniformly ^13^C-labelled phenanthrene ([U-^13^C]phenanthrene), following previous protocols^72,73^. Unlabelled phenanthrene (99.5% purity) and [U-^13^C]phenanthrene were from Sigma-Aldrich (Sigma, Dorset, UK). Briefly, a known mass of the compound (unlabelled or [U-^13^C]-labelled was dissolved in acetonitrile in a vented glass container and left for 48 h to allow the solvent to evaporate. To the residual crystallised phenanthrene, sdH_2_0 was added to produce a final aqueous phenanthrene solution of 1.1 mg L^−1^, which was then used to prime the nanoplastic particles. For this, 1 mg each of the nanoplastic sizes (500 nm or 1000 nm) was added to 10 mL of the prepared unlabelled or [U-^13^C]-labelled phenanthrene solution and allowed to incubate for 24 h with gentle rotatory shaking (~ 80 rpm) at 21 °C in the dark. The plastic particles were recovered by centrifugation (10,000×*g* for 4 h at 20 °C) and resuspended in sdH_2_O. The nanoplastic suspensions with the unlabelled compounds were used for the agglomerate experiments, whereas the nanoplastic suspensions with the [U-^13^C]-labelled compound were used for the SIP experiment (described below). Before using these nanoplastic suspensions in their respective experiments, sub-samples were taken to re-measure and confirm the size and ζ-potential of the particles. For the 500 nm plastic particles, adsorption of the [U-^13^C]phenanthrene was examined by visual inspection using a Zeiss (Axio Scope.A1) epifluorescence microscope fitted with a Zeiss digital fluorescence imaging camera (AxioCam MRm) with excitation and emission wavelengths at 250 nm and 366 nm, respectively^74^. In addition, sub-samples of the [U-^13^C]phenanthrene solution were taken prior to the addition of the plastic particles, and also after their removal, in order to determine phenanthrene concentrations (see below) as further confirmation that phenanthrene had become adsorbed onto the surface of the 500 nm nanoplastic particles.

### Nanoplastic agglomerate formation experiments

The 500 nm and 1000 nm nanoplastic particles sorbed with or without unlabelled phenanthrene were evaluated for their ability to form agglomerates in the natural seawater collected from coastal water in the Firth of Forth. For this, 37.5 mL of the seawater was added to twelve 40-mL scintillation vials. Three of the vials were supplemented with 500 nm PS plastic particles with adsorbed phenanthrene, three with the 500 nm plastic particles without adsorbed phenanthrene, another three with the 1000 nm plastic particles with adsorbed phenanthrene, and another three with the 1000 nm particles without adsorbed phenanthrene. Each of the two nanoplastic particle sizes was added by first preparing stock suspensions to a concentration of 10 mg mL^−1^ in sdH_2_O (with no other additives), of which 20 μL was added to the respective scintillation vials. The final concentration of the plastic particles added to each vial was 5 µg mL^−1^. This equated to approximately 4.6 × 10^7^ particles per mL for vials containing 500 nm PS particles, and 3.7 × 10^6^ of the 1000 nm PS particles per mL. All experimental manipulations were carried out aseptically and each treatment was performed in triplicate. Controls (no added plastic particles or phenanthrene) were also prepared (in triplicate) and incubated in exactly the same way. All vials were hermetically sealed, leaving approximately a 2 mL headspace, and placed onto a low-profile roller table with constant gentle rotation (~ 15–20 rpm) for 7 days at 10 °C, which was the *in-situ* temperature at the location where the water sample was collected. This type of experimental setup maintains these incubations in a constant gentle turbulence to simulate conditions near the sea surface and has been used in similar studies investigating plastics agglomeration^19^ and marine snow formation^19,75^.

At the termination of these incubations, the volume from each of the vials was passaged through 100 μm celltrics filters (Sysmex, Germany) to isolate any agglomerates that formed. Agglomerates collected on filters from each treatment were transferred to sterile 2 mL micro-centrifuge tubes for total DNA extraction; this was done for agglomerates collected from each replicate of each treatment.

### SIP experiment

To identify members of the bacterial community capable of metabolising plastic-bound phenanthrene, a SIP experiment was conducted using [U-^13^C]phenanthrene adsorbed onto the 500 nm PS particles (prepared as described above). For this experiment, all glassware used had been pre-baked at 550 °C for two hours to completely burn off any plastic residues that may have been present inside the vials and inner lining of the caps. The vials and caps were further cleaned with 5% nitric acid followed by an acetone wash, then thoroughly rinsed with distilled water and finally autoclaved prior to use. The vials used were amber in colour to prevent potential light-induced effects, as aromatic hydrocarbons, like phenanthrene, are susceptible to photolysis.

When it comes to the design and execution of SIP, careful attention must be employed in order for it to yield interpretable and unambiguous results. A major challenge in SIP is obtaining sufficient incorporation of the ^13^C into biomass, in which case for DNA-SIP this relates to its enrichment into DNA. Whilst the extent of labeling can be increased with longer incubation times, this can lead to the ^13^C becoming distributed among other members of the microbial community that may not directly be capable of metabolizing the isotopically-labeled substrate—i.e. by cross-feeding on ^13^C-labeled metabolic byproducts, intermediates, or dead cells^76^. To avert this, we set up triplicate incubations with the unlabelled hydrocarbon in order to tractably measure the degradation (by gas chromatography mass spectrometry (GC–MS); described below) of the phenanthrene to help guide our selection of the point at which to terminate the ^13^C incubations (endpoint of experiment) whereby sufficient ^13^C incorporation had been achieved with minimal cross-feeding. For SIP, we set up duplicate incubations supplemented with the 500 nm plastic particles primed with [U-^13^C]phenanthrene. Triplicate incubations of acid-killed controls (pH < 2) containing unlabelled phenanthrene were also prepared by adding ca. 700 µL of 85% phosphoric acid to take into account any disappearance of the hydrocarbon due to abiotic factors. All incubations were performed in 40-mL scintillation vials, with the seawater and nanoplastics added as described above for preparation of the agglomerate formation experiments. At the endpoint of the experiment (determined to be 7 days), the agglomerates were harvested from the paired flasks amended with the [U-^13^C]phenanthrene, and the filtrate (water fractions) from these incubations was passaged through sterile 0.22-µm filters to collect the free-living microbial biomass. Genomic DNA from the agglomerates and free-living community was extracted using the method of Tillett and Neilan^77^] for subsequent microbial community analysis (described below).

Cesium chloride gradient ultracentrifugation and identification of ^13^C-enriched DNA was performed as previously described^78^. The ^13^C-enriched heavy DNA fractions were selected based on evidence from denaturing gradient gel electrophoresis (DGGE). The heavy DNA from each of the SIP incubations was sequenced using Oxford Nanopore Technologies Minion sequencing, as described below.

### DNA extraction and 16S rRNA gene amplicon sequencing

For the extraction of DNA from filters, the filters were immersed in liquid nitrogen and milled to form a powder, and then genomic DNA was extracted using the method of Tillett and Neilan^77^. For harvested agglomerates, DNA was extracted in the same way but bypassing the use of the liquid nitrogen and milling treatment. Extracted DNA was stored at − 80 °C for microbial community sequencing, as described below.

Illumina MiSeq paired-end sequencing was performed to analyze the bacterial community of the samples from the agglomerate experiment. For this, genomic DNA was subject to PCR amplification of the V4 hypervariable region of the 16S rRNA gene using oligonucleotides 515F-806R^79^ inclusive of MiSeq overhangs and golay barcodes. Reaction volumes for each PCR reaction (25 μL total volume) were as follows: 10 μL of PCR mastermix (Sigma Jumpstart hot-start PCR mix); 0.5 μL (10 μM) of forward primer; 0.5 μL (10 μM) of reverse primer; 1 μL of template DNA. Thermocycler conditions were: 94 °C for 180 s initial denaturation; 35 cycles of 94 °C for 45 s, 53 °C for 50 s, 72 °C for 90 s; final elongation at 72 °C for 10 min. PCR product purification was conducted using Exonuclease and FastAP to remove primers and nucleic acids which had not annealed, and to dephosphorylate the samples prior to sequencing. This was achieved by adding PCR product (20.0 µL), Exonuclease I (0.5 µL) and FastAP (1.0 µL) to a 25 µL reaction and heating to 37 °C for 45 min, before inactivating at 85 °C for 15 min. Illumina MiSeq paired-end sequencing was conducted at the Singapore Centre for Environmental Life Sciences Engineering.

For the ^13^C-enriched 'heavy' DNA from SIP, the bacterial community was analysed using Oxford Nanopore long read sequencing of the full length 16S rRNA gene using the ONT sequencing kit #SQK-16S024 (Oxford, UK). In brief, labelled nucleic acids from the heavy fractions were amplified using barcoded 27f and 1492r primers from the ONT kit, as per the manufacturer’s instructions. The resulting amplicons were pooled together in an equimolar mix and sequenced using a R9 ONT flowcell for 48 h.

### Bioinformatic analysis

Processing of the amplicon sequence data from Illumina sequencing was performed using the DADA2 package as wrapped in QIIME2^80^. In brief, paired end Illumina reads were combined to form contiguous sequences. A fragment cut-off of 220 bp was established to maintain quality. These contigs were examined for low quality phred scores and any identified chimeric sequences were removed. All quality-approved sequences were compared on a single nucleotide resolution and the resulting single nucleotide variants (SNVs) were identified using the Green Genes database of 16S rRNA gene taxonomy. For *alpha*-diversity rarefaction analysis, sampling was standardized to 7000 sequences per sample.

All reads obtained using the Oxford Nanopore long read platform were basecalled using Guppy (v3.3.0)^81^ and adapters removed using porechop (v0.2.4)^82^. All reads were quality filtered (NanoFilt, v2.7.1)^83^ to include only those reads with a QC score > 10 and a minimum length of 1300 bases. The identification of each read was performed by comparison to the Silva 16S database (v138) using Kraken2 package (v2.0.8-beta)^84^.

All sequences from the agglomerate and SIP experiments were deposited in the SRA repository under accession numbers SAMN11998198 to SAMN11998266.

### Quantification of phenanthrene concentrations

Samples (2 mL) for quantification of phenanthrene were each extracted three times with dichloromethane:methanol (9:1 v/v). Solvent extracts were evaporated to dryness under a gentle stream of high-purity nitrogen and the resultant residue reconstituted in 25 μL hexane. Phenanthrene was detected and quantified using a ThermoScientific TRACE 1300 gas chromatograph coupled to a dual ISQ LT mass-selective detector and flame ionization detector (GC/MS-FID). For this, reconstituted extracts were injected by programmed temperature vaporization (PTV) in splitless mode at 300 °C onto a DB-5 ms column (30 m × 0.25 mm inner diameter × 0.25 μm film thickness). The oven was at first held for 2 min at 60 °C, then ramped at 4 °C/min to 300 °C and held for an additional 15 min. Samples were run both in selective ion mode (SIM) and full scan (total ion count [TIC]) mode. Compounds were identified through full scan mass spectra in conjunction with retention time against authentic polycyclic aromatic hydrocarbon (PAH) standards (QTM PAH Mix [CRM47930]). Phenanthrene was additionally quantified in SIM (*m*/*z* 178) through response factors determined from 5-point concentration calibration curve of identical PAH standards.

### Statistical analyses

All statistical analyses were conducted in R v3.4.1^85^ using the vegan package v2.5-4^86^. All multi-variate comparisons were conducted using Permanova. For samples taken from incubations that used phenanthrene attached to the plastic particles, the Permanova was nested, thus eliminating any independence. Comparisons of individual taxa for each treatment type were conducted using an Analysis of Variance (ANOVA) test. Shannon-Weiner diversity indices were calculated on untransformed data to determine sample α-diversity. NMDS ordinations display β-diversity. All data was log transformed to meet the assumptions of parametric analyses as required.

### Supplementary Information

Supplementary Legends.

Supplementary Table S1.

Supplementary Table S2.

## Data Availability

All data used and/or analyzed during the current study are presented in the article. All 16S rRNA gene sequence data has been deposited in the SRA repository under Accession Numbers SAMN11998198 to SAMN11998266.
