# Supplementary material for: Identification of the bacterial community that degrades phenanthrene sorbed to polystyrene nanoplastics using DNA-based stable isotope probing
Source: Sci Rep. 2024 Mar 4;14:5229. doi: 10.1038/s41598-024-55825-9 (PMC10909871; doi:10.1038/s41598-024-55825-9)
Supplement: Supplementary file 1 — Supplementary Legends. [file 41598_2024_55825_MOESM1_ESM.docx]

**Supporting Information**

**Table S1.** SIP-identified taxa and their relative abundance from ^13^C-enriched DNA recovered from agglomerates (AH1, AH2) and the water surrounding them (WH1, WH2) in the experiments with coastal water from the Firth of Forth supplemented with 500 nm polystyrene nanoplastics adsorbed with [U-^13^C]phenanthrene.

**Table S2.** Relative abundance of the various taxa identified in the ^13^C-enriched community compared to those found in the ^12^C-unlabelled community of the nanoplastic agglomerates and surrounding seawater. Taxa highlighted in green represent those detected in the ^13^C-enriched DNA fractions by at least 1% abundance from agglomerates, and also their % abundance is at least 5-fold higher enriched in one or both of the heavy fractions compared to in the corresponding duplicate ^12^C-unlabelled fractions.
