# Supplementary material for: Identification of the bacterial community that degrades phenanthrene sorbed to polystyrene nanoplastics using DNA-based stable isotope probing
Source: Sci Rep. 2024 Mar 4;14:5229. doi: 10.1038/s41598-024-55825-9 (PMC10909871; doi:10.1038/s41598-024-55825-9)
Supplement: Supplementary file 2 — Supplementary Table S1. [file 41598_2024_55825_MOESM2_ESM.docx]

**Supporting Information**

**Table S1.** SIP-identified taxa and their relative abundance from ^13^C-enriched DNA recovered from agglomerates (AH1, AH2) and the water surrounding them (WH1, WH2) in the experiments with coastal water from the Firth of Forth supplemented with 500 nm polystyrene nanoplastics adsorbed with [U-^13^C]phenanthrene.
